# Novel derivatives of eugenol as potent anti-inflammatory agents *via* PPARγ agonism: rational design, synthesis, analysis, PPARγ protein binding assay and computational studies†

**DOI:** 10.1039/d2ra02116a

**Published:** 2022-06-07

**Authors:** Noor Fathima Anjum, Dhivya Shanmugarajan, Vasanth Kumar Shivaraju, Syed Faizan, Namburu Lalitha Naishima, B. R. Prashantha Kumar, Saleem Javid, Madhusudan N. Purohit

**Affiliations:** Department of Pharmaceutical Chemistry, Farooqia College of Pharmacy Mysuru 570 015 India; Department of Pharmaceutical Chemistry, JSS College of Pharmacy Mysuru 570 015 India madhusudhanpurohit@jssuni.edu.in +91-821-2548359 +91-821-2548353; JSS Academy of Higher Education & Research Mysuru 570 015 India; DOS in Chemistry, Karnataka State Open University Mukthagangothri Mysuru 570 006 India

## Abstract

Eugenol is a natural product abundantly found in clove buds known for its pharmacological activities such as anti-inflammatory, antidiabetic, antioxidant, and anticancer activities. It is well known from the literature that peroxisome proliferator-activated receptors (PPARγ) have been reported to regulate inflammatory responses. In this backdrop, we rationally designed semi-synthetic derivatives of eugenol with the aid of computational studies, and synthesized, purified, and analyzed four eugenol derivatives as PPARγ agonists. Compounds were screened for PPARγ protein binding by time-resolved fluorescence (TR-FRET) assay. The biochemical assay results were favorable for 1C which exhibited significant binding affinity with an IC_50_ value of 10.65 μM as compared to the standard pioglitazone with an IC_50_ value of 1.052 μM. In addition to the protein binding studies, as a functional assay, the synthesized eugenol derivatives were screened for *in vitro* anti-inflammatory activity at concentrations ranging from 6.25 μM to 400 μM. Among the four compounds tested 1C shows reasonably good anti-inflammatory activity with an IC_50_ value of 133.8 μM compared to a standard diclofenac sodium IC_50_ value of 54.32 μM. Structure–activity relationships are derived based on computational studies. Additionally, molecular dynamics simulations were performed to examine the stability of the protein–ligand complex, the dynamic behavior, and the binding affinity of newly synthesized molecules. Altogether, we identified novel eugenol derivatives as potential anti-inflammatory agents *via* PPARγ agonism.

## Abstract 2

Rational design, synthesis, analysis, PPARγ protein binding assay and computational studies of novel eugenol derivatives.

## Graphical abstract

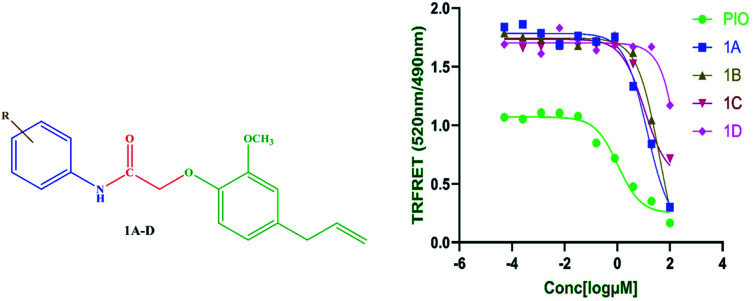

## 1. Introduction

**Fig. 1:**
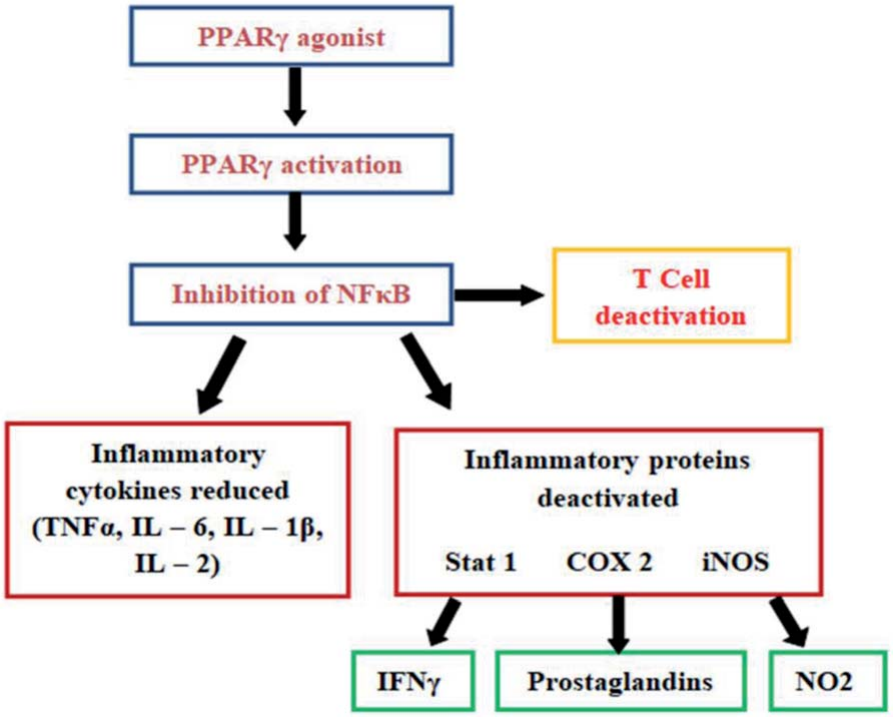
Activated PPARγ inhibits the activity of NF-κB which is responsible for inflammatory reactions.

Peroxisome proliferator-activated receptors (PPARs) are well-characterized type II nuclear receptors and transcription factors. PPARs represent a group of three receptors PPARα, PPARβ/δ, and PPARγ which causes inhibition of NF-κB activation as depicted in (Fig. 1).^1^ Nuclear factor kappa-B (NF-κB) signaling is an important part of the immune response as it plays an important role in inflammatory processes by inhibiting factor-α (TNF-α), interleukin 1β (IL-1β), interleukin 6 (IL-6) and nitric oxide. The target protein, PPARγ, is reported to regulate inflammatory responses in physiological systems.^2,3^

The process of inflammation is a multifaceted protective response of vascular tissues to hazardous substances such as microbes, irritants, and damaged cells.^4^ Inflammation is caused by the upregulation of proinflammatory cytokines and chemokines, which activate and interact with a wide range of immune factors. Macrophages and monocytes play a key role in the inflammatory process by secreting inflammatory cytokines, such as TNFα and IL-6, as well as by releasing nitric oxide (NO) from inducible nitric oxide synthase (iNOS).^5–7^

2-Methoxy-4-(2-propenyl) phenol, also known as eugenol, from the class of phenylpropanoids is an allylbenzenes compound. It is the main constituent of the spice *Syzygium aromaticum* (also known as *Eugenia caryophyllata*), from the family Myrtaceae. Eugenol is a pharmacologically active substance found in plant essential oils. It can also be found in cinnamon (*Cinnamomum verum*), basil (*Ocimum basilicum* L.). Plants like *Myristica fragrans* (Nutmeg), *C. loureirii nees* (Saigan Cinnamon), *Ocimum tenuiflorum* (Tulsi), *Illicium anisatum* (Star Anise), *Melissa officinalis* (Lemon Balm) also contain eugenol. Eugenol is said to possess various biological properties like antiseptic, analgesic, and antibacterial properties.^8–13^ Besides its potential applications in treating some diseases, it has been studied in terms of its anti-inflammatory, antioxidant, antispasmodic, antidepressive, antigenotoxic, and anticarcinogenic properties.^14–16^

Further studies are needed to determine the signal transduction pathways that are blocked and activated by the immune system to determine how eugenol inhibits inflammation. It has been reported that eugenol inhibits the phosphorylation of the Raf/MEK/ERK1/2/p47phox pathway, thereby inhibiting the generation of superoxide anion in neutrophils. Several studies have shown that eugenol inhibits pro-inflammatory mediators including interleukins IL-1β and IL-6, tumor necrosis factor-alpha (TNF-α) and prostaglandin E2 (PGE2), inducible expression of oxide nitric synthase (iNOS) and activation of cyclooxygenase-2 (COX-2), nuclear factor kappa B (NF-κB), leukotriene C4 and 5-lipoxygenase (5-LOX).^17–19^

In this backdrop, we aimed at developing new eugenol derivatives to study their potential anti-inflammatory activity. Therefore, we rationally designed eugenol based PPARγ agonists, synthesized, purified, analyzed, screened for PPARγ binding, and evaluated for *in vitro* anti-inflammatory activity. Molecular docking, SAR analysis, and Molecular Dynamic simulations were also carried out to correlate the results.

## 2. Results and discussion

### 2.1 Design of eugenol derivatives as PPARγ agonists

**Fig. 2:**
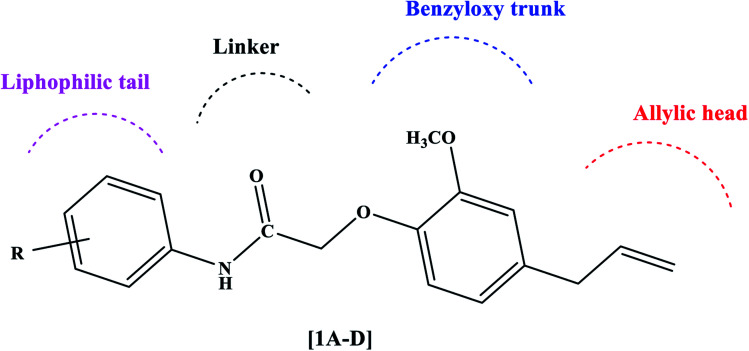
Rational design of eugenol derivatives as PPARγ agonists.

The PPARγ agonists possess structural features such as a heterocyclic head usually thiazolidinedione followed by a benzyloxy trunk, two carbon linkers, and a lipophilic tail. Considering these structural features, we designed novel eugenol derivatives that possess a benzyloxy trunk, two carbon linkers, and a lipophilic tail (Fig. 2). However, the heterocyclic head is replaced with an aliphatic chain with a terminal double bond. This was the only difference between existing PPARγ agonists and the present eugenol derivatives.

### 2.2 Synthesis

**Scheme 1:**
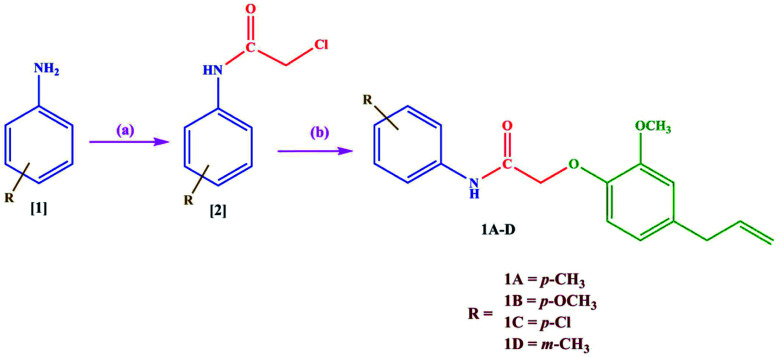
Synthesis of new eugenol derivatives: (a) dichloromethane, chloroacetyl chloride, triethylamine, 0–5 °C to rt stirred for 10 h (b) eugenol, acetone, K_2_CO_3,_ 45 °C stirred for 26 h.

The acylated amines 2 were synthesized by treating substituted aromatic amines 1 with chloroacetyl chloride using dichloromethane as a solvent. The acylated amines were then linked to eugenol in presence of anhydrous potassium carbonate and dry acetone to obtain novel eugenol derivatives^20^ (1A–D) (Scheme 1). The characterization of the synthesized compounds was done by IR, ^1^H-NMR, ^13^C-NMR, and mass spectrometry. The melting points of the synthesized derivatives were determined in open capillaries using melting point apparatus and are uncorrected. TLC was performed using *n*-hexane and ethyl acetate in the ratio of (8 : 2) as mobile phase on aluminium plates which are precoated with silica gel G. Compounds were named following the IUPAC rules. The IR spectra were obtained using a KBr pellet technique on a Shimadzu infrared FTIR spectrophotometer and are given in cm^1^. The IR spectrum shows the disappearance of the band attributed to the –OH group at around 3250 cm^−1^. The band at 3460 cm^−1^ indicates the presence of the (NH) group and the presence of (C=O) at 1690 cm^−1^. The solvent used for ^1^H-NMR and ^13^C-NMR was CDCl_3_. The chemical shifts are quantified in parts per million (*δ* ppm), with the notation s = singlet, d = doublet, t = triplet, and m = multiplet. The ^1^H-NMR spectra of all the newly synthesized compounds exhibit a singlet at around *δ* 8.90–9.00 ppm which is attributed to the characteristic of NH in addition to aromatic protons. One proton of CH= gives multiplet at around *δ* 5.9–6.0 ppm and two protons of =CH_2_ give doublet at around *δ* 5.1 ppm. Similarly, all the ^13^C NMR spectra show distinguishing signals *δ* 166.7–172.1 ppm due to the ketonic carbon, in addition to other normal signals of carbon. The molecular mass of all the final compounds was determined by LC-MSMS. All these facts confirm the formation of final compounds (1A–D) Thus, the spectral data confirm the presented structures of all newly synthesized eugenol derivatives.

### 2.3 ADMET and TOPKAT profile

ADMET and toxic prediction of the compounds is essential parameters that need to be assessed the efficacies or risks of small compounds.^21^ The pharmacokinetics and dynamic profiles of synthesized compounds are tabulated (Table 1). The potential drug must cross certain barriers in the biological system to show ideal ADME properties. Hence, it was qualitatively assessed for the eugenol derivatives. Furthermore, the analysis depicted all the compounds obey the oral drug-likeness Pfizer rule and are free from mutagenicity and carcinogenicity^22^ as tabulated in (Tables 1 and 2).

**Table 1:** ADMET and toxicity profile of designed PPARγ agonists^a^

PPARγ agonist	Solubility	BBB	CPY2D6	HIA	NTP_RAT		Ames mutagen
					Male	Female	
1A	2	1	NI	0	NC	NC	NM
1B	3	1	NI	0	NC	NC	NM
1C	2	1	NI	0	NC	NC	NM
1D	2	1	NI	0	NC	NC	NM
Pioglitazone	2	1	NI	0	NC	NC	NM

a NI: non-inhibitor, NC: non-carcinogen, NM: non-mutagen.

**Table 2:** Pfizer rule of 5 and set dosage range of rat model^a^

PPARγ agonist	Alogp	MW	HBA	HBD	Rat_Oral LD_50_ g per kg_body_weight	Rat_Inhalational LC_50_ mg m^−3^ h^−1^	Carcinogenic Potency TD_50__Rat mg per kg_body_weight per day
1A	3.927	311.375	4	1	6.38057	10 950.5	43.3141
1B	3.927	327.374	4	1	4.47735	10 394	114.334
1C	3.425	331.793	5	1	5.06277	6740.29	49.4299
1D	4.105	311.375	4	1	4.10077	7127.09	34.3322
Pioglitazone	3.907	356.439	5	1	7.2814	6054.58	61.7341

a MW: molecular weight, HBA: hydrogen bond acceptor, HBD: hydrogen bond donor.

### 2.4 Structure-based drug designing

**Fig. 3:**
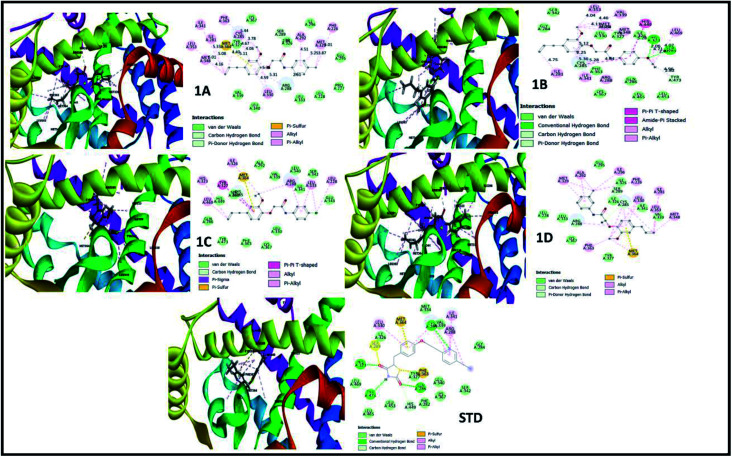
3D and 2D interaction analysis diagram of synthesized compounds (1A, 1B, 1C, 1D) and pioglitazone (STD) with receptor protein (PDB_ID: 2PRG).

The perception of structure-based drug designing was implemented for known compounds and drug target proteins. For PPAR-gamma receptor–ligand interaction in the ligand-binding domain region as Phe282, Cys285, Gln286, Arg288, His323, Tyr327, Leu330, Gly338, His449, and Leu469 is favorable for eugenol derivatives. Further, the binding energies calculated using the implicit solvent model PBSA are tabulated (Table 3). During interaction probing it was observed that eugenol derivatives interact with the one or more amino acid residues of the ligand-binding domain of PPAR-gamma. The amino acids Arg288, Ser289, and His323 interact to form hydrogen bond interactions (Fig. 3) and other amino acids are favorable for hydrophobic interactions. Besides, binding energies are significantly higher with 1C and standard as compared with other complexes.

**Table 3:** Binding energies calculated using implicit solvent model PBSA and CDOCKER docked energy^a^

Compounds	PBSA binding energy in kcal mol^−1^		CDOCKER energy	
	PPAR-gamma	Lysozyme	PPAR-gamma	Lysozyme
1A	−15.026	−1.176	−20.8756	−15.1195
1B	−5.5013	−1.013	−14.2636	−13.1921
1C	−20.7787	−1.952	−21.1956	−16.7432
1D	−13.9708	−1.342	−17.3625	−15.2079
STD	−25.5827	−2.294	−22.891	−19.687

a Pioglitazone for PPAR-gamma and diclofenac for lysozyme.

### 2.5 Structure–activity relationship (SAR)

A structure–activity relationship (SAR) is a key tool and information pool in organic chemistry *en routes* to synthesize many compounds for the better and more potent acquisition of compounds for various drug targets.^23^ It is a guide used to predict the compound's biological activities from chemical structure, the functional moieties attached to the compounds play a significant role. In this study receptor–ligands complex of 1A–1D was used for interaction pharmacophore analysis as shown in (Fig. 5). Interestingly, the 4-allyl-2-methoxyphenoxy group is common in all the compounds sharing two hydrophobic and one aromatic hydrophobic. The potential high active compound 1C (IC_50_ = 10.65 μM) possesses halogen group (cl) in the *para* position of benzene enhances biological activity. But, OCH_3_ substitution in the *para* position reduces the biological activity in compound 1B. Hence the position of the parafunctional group is considered a crucial pharmacophore for compounds to influence the PPAR gamma protein binding and is directly proportional to the biological activity (Fig. 6).

**Fig. 4:**
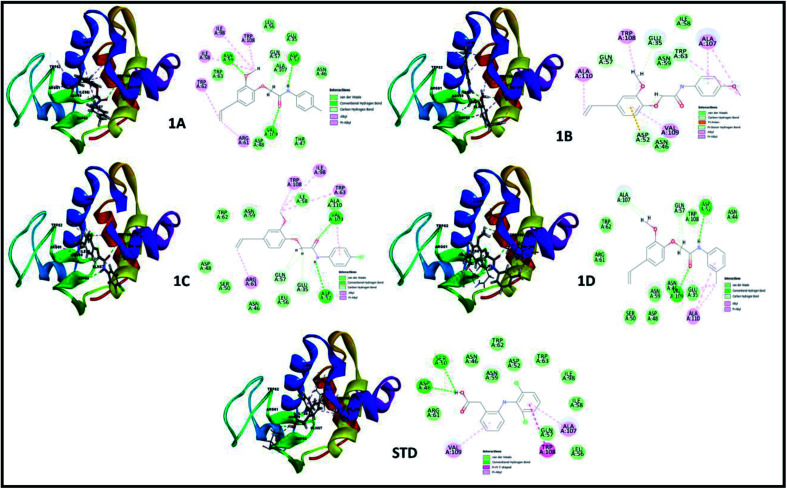
3D and 2D interaction analysis diagram of synthesized compounds (1A, 1B, 1C, 1D) and diclofenac (STD) with lysozyme egg protein (PDB_ID: 3WXU).

In both drug targets, many van der Walls interactions offered by residues within 5 Å and molecular docking of lysozyme egg protein with eugenol derivatives have a tendency to form hydrogen and hydrophobic interactions (Fig. 4).

**Fig. 5:**
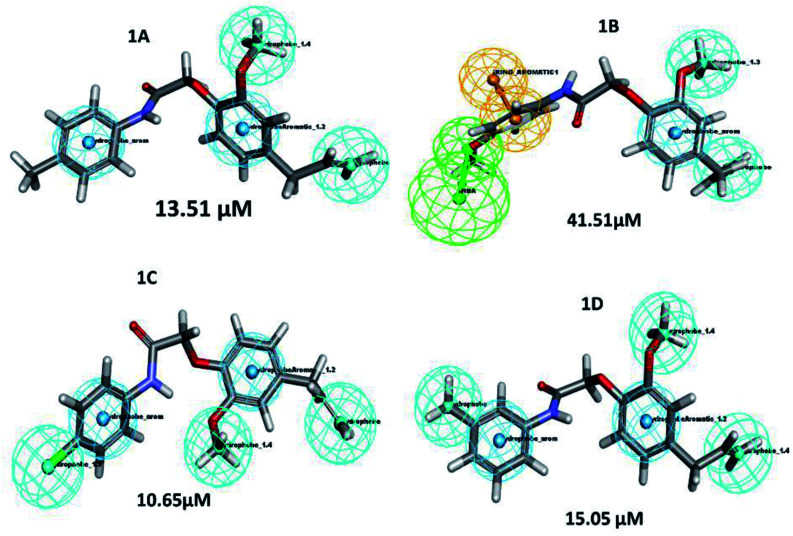
Pharmacophore features represent the structure–activity relationship of synthesized compounds that are screened against PPAR gamma.

**Fig. 6:**
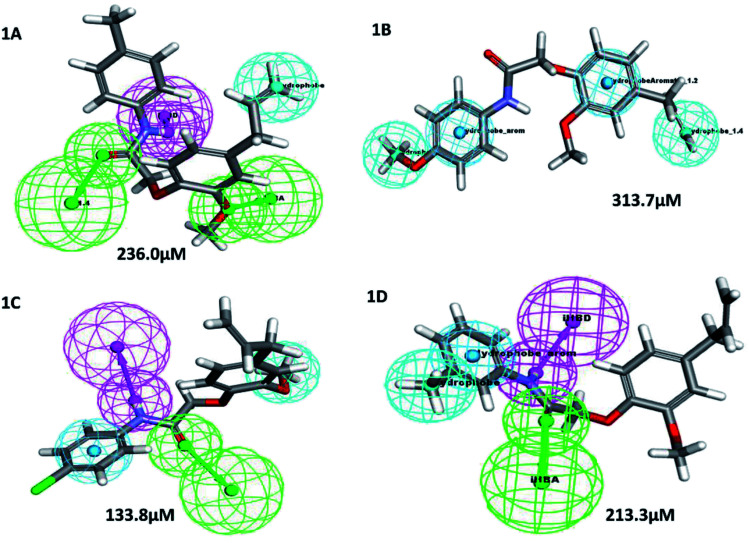
Pharmacophore features represent the structure–activity relationship of newly synthesized compounds pharmacophore with lysozyme. *Color-coded of pharmacophore: green sphere represents hydrogen bond acceptor (HBA), cyan sphere represents the hydrophobic region and magenta sphere represents hydrogen bond donor (HBD), orange color represents ring aromatic (RA).

Similarly, the lysozyme enzyme interaction complex was probed for SAR analysis depicted (Fig. 6) the halogen, chlorine attached at the *para* position of the benzene ring shows good biological activity than other eugenol derivatives. Substitution of OCH_3_ (1B) or CH_3_ (1A) reduced the biological activity. Whereas, CH_3_ at the *meta* position of the benzene ring in compound 1D shows better activity than 1B and 1A. Thus, SAR analysis shows the importance of the functional moiety pharmacophore of the compounds.

### 2.6 Time-dependent parameter conformational analysis of the complex

**Fig. 7:**
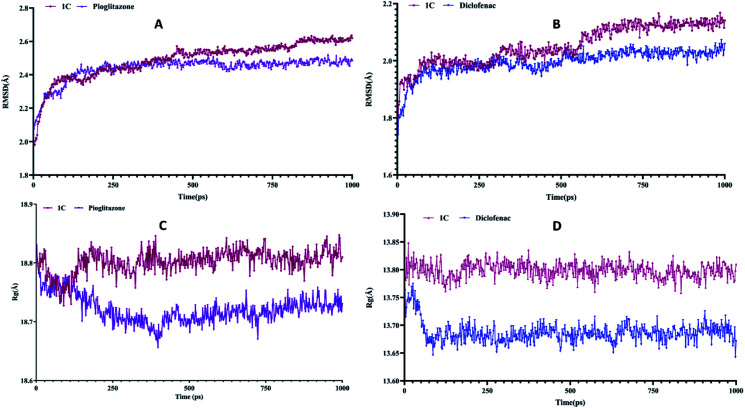
(A) and (B) is the root mean square deviation (RMSD) of PPARγ and lysozyme and (C) and (D) is the radius of gyration (*R*_g_) of PPAR-gamma and lysozyme compared with standard.

The binding modes of best-docked molecules of receptor–ligand were further studied using a molecular dynamics simulation study for a simulation time of 1000 ps.^24^ The geometric features of the protein–ligand complexes, such as root mean square deviation (RMSD) and radius of gyration (*R*_g_), were determined to assess the system's stability. The RMSD is used to measure the root mean square deviation of atomic positions of each conformation. The average distance between the atoms of various structural conformations of protein and ligand over a period of time. The average RMSD of the Cα atoms of peroxisome proliferator-activated receptor (PPAR) gamma and heavy atoms of 1C was determined to be 2.490 ± 0.1105 Å. In contrast, the standard drug pioglitazone with peroxisome proliferator-activated receptor (PPAR) gamma complex had an average RMSD of 2.440 ± 0.07039 Å (Fig. 7A). Similar analysis of lysozyme bound complex average root mean square deviations are 2.054 ± 0.06697 Å and 1.994 ± 0.04284 of 1C and standard drug (diclofenac) respectively (Fig. 7B). Hence, a conformational change of the compounds has a direct influence on biological activity. *R*_g_ is the root mean square distance between each atom in a system and its center of mass. The *R*_g_ values for protein–ligand complexes: peroxisome proliferator-activated receptor (PPAR) gamma 1C and pioglitazone show fluctuations between 18.6 Å to 18.9 Å while, lysozyme bound complexes *R*_g_ values are between 13.65 Å to 13.85 Å. The energy parameter analysis of all the complexes shows fewer variations (Table 4) which indicates energetically all three complexes are good. Overall, peroxisome proliferator-activated receptor (PPAR) gamma 1D complex shows less deviations with time-dependent parameter analysis and 1C lysozyme complex shows best in inflammatory activity.

**Table 4:** Energy parameter analysis values and deviations

Energy parameters	1C	Pioglitazone	1C	Diclofenac
Potential	−16728 ± 166.9	−1668 ± 128.7	−7403 ± 57.74	−7454 ± 39.93
Kinetic	3324 ± 31.60	3326 ± 34.97	1502 ± 20.45	1488 ± 20.59
Electrostatic	−20010 ± 161.1	−1993 ± 121.6	−8829 ± 64.48	−8909 ± 39.20
van der Waals	−1659 ± 27.25	−1623 ± 26.76	−742.4 ± 16.75	−734.7 ± 17.77

### 2.7 TR-FRET assay

**Fig. 8:**
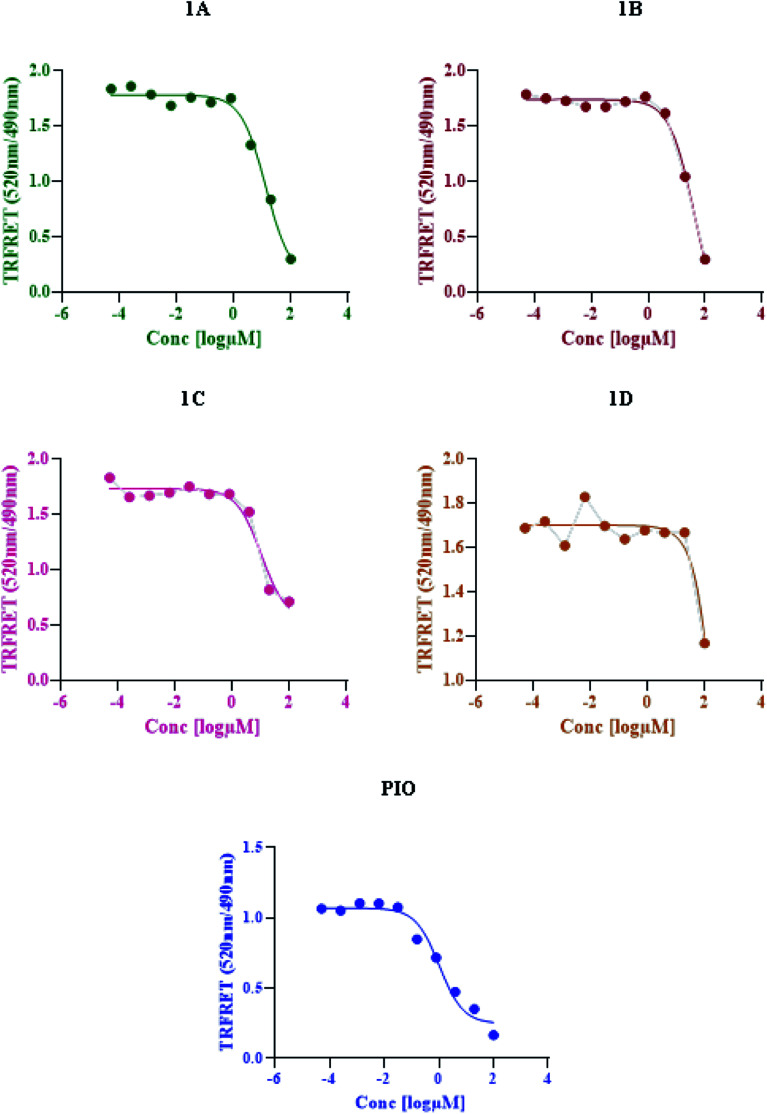
LanthaScreen TR-FRET PPARγ protein binding assay: a comparison of synthesized compounds and standard pioglitazone.

Based on time-resolved fluorescence resonance energy transfer (TR-FRET), the nuclear receptor competitive binding assay was utilized to identify PPARγ ligands. When a fluorescent ligand (tracer) is bound to the receptor, energy transfer from the antibody to the tracer occurs, and a high 520/495 ratio is detected.^25^ The tracer is displaced from PPARγ-LBD by a chemical under test, resulting in a decrease in the FRET signal and a low TR-FRET ratio (Fig. 8). The assay results indicate that 1C shows an IC_50_ value of 10.65 μM whereas standard pioglitazone shows an IC_50_ value of 1.052 μM. This suggests that 1C has a binding affinity for the target protein that is similar to that of typical pioglitazone. The IC_50_ values of standard drug pioglitazone and synthesized compounds are tabulated in (Table 5).

**Table 5:** IC_50_ values of standard pioglitazone and synthesized compounds

S. no.	Compound	IC_50_ (μM)
1	Pioglitazone	1.052
2	1A	13.51
3	1B	41.51
4	1C	10.65
5	1D	15.05

### 2.8 Statistical analysis

All the results were expressed as means ± standard deviation (SD) and the data were analyzed using one-way ANOVA using Graph pad prism 8.0 software. *P* values < 0.05 were considered significant.

### 2.9 *In vitro* anti-inflammatory activity

#### 2.9.1. Albumin denaturation assay

The protein denaturation has long been acknowledged as a factor of inflammation. Inflammatory diseases and disorders such as diabetes, rheumatoid arthritis, and cancer are associated with the denaturation of protein. The ability of a substance to prevent protein denaturation helps to prevent inflammatory disorders.^26^ This assay finds its importance as part of preclinical studies to establish the potency and efficacy of the new molecules in the process of drug discovery.^27–29^

**Fig. 9:**
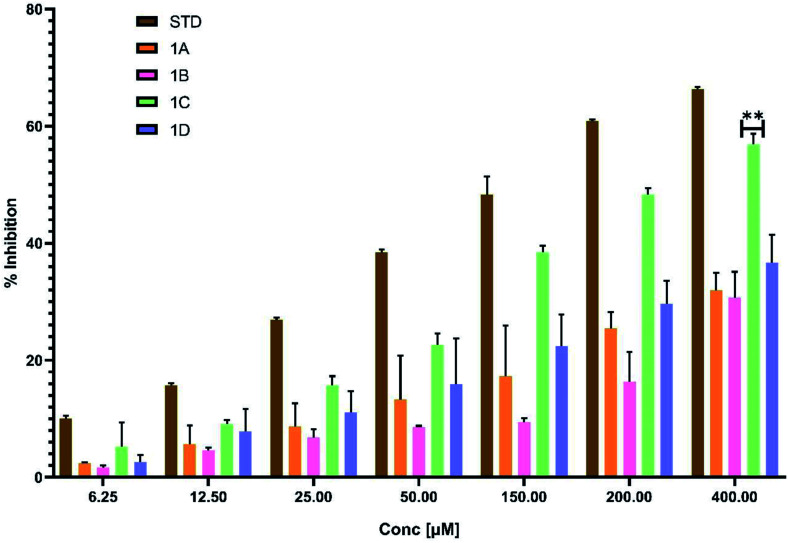
Effect of synthesized compounds and standard diclofenac on albumin denaturation values were expressed as means ± SD (*n* = 3); **p < 0.05 *vs.* Std.

In this assay egg albumin is used as protein. Protein denaturation is achieved by keeping the reaction mixture at 70 °C in a water bath for 10 minutes. As a part of the investigation of the mechanism of the anti-inflammatory activity, the ability of the synthesized compounds and standard diclofenac to inhibit protein denaturation was studied.^30–33^ At various concentrations, it proved efficient in preventing heat-induced albumin denaturation, as shown in (Table 6). The novel synthesized compounds significantly (*p* < 0.05) inhibited the albumin denaturation was shown in (Fig. 9) and maximum inhibition of 48.08 ± 1.143 at 200 μM was observed for 1C when compared with standard diclofenac 48.28 ± 3.139 at 150 μM. IC_50_ values of standard diclofenac and synthesized compounds on inhibition of albumin denaturation were statistically significant (Table 7 and Fig. 9).

**Table 6:** Effect of percentage inhibition of albumin denaturation at different concentrations with statistical significance at *p* < 0.05 means ± SD of triplicate trials for each concentration^a^

Concentration in [μM]	Standard diclofenac	1A	1B	1C	1D
6.25	10.03 ± 0.463	2.42 ± 0.165	1.713 ± 0.344	5.23 ± 4.116	2.66 ± 1.123
12.5	15.68 ± 0.429	5.70 ± 3.186	4.57 ± 0.540	9.14 ± 0.615	7.88 ± 3.800
25	26.96 ± 0.336	8.72 ± 3.934	6.82 ± 1.371	15.69 ± 1.590	11.08 ± 3.650
50	38.48 ± 0.468	13.30 ± 7.511	8.59 ± 0.205	22.613 ± 1.980	15.92 ± 7.793
150	48.28 ± 3.139	17.24 ± 8.711	9.45 ± 0.681	38.48 ± 1.105	22.43 ± 5.426
200	60.90 ± 0.223	25.41 ± 2.869	16.35 ± 5.087	48.08 ± 1.143	29.65 ± 3.941
400	66.29 ± 0.375	31.97 ± 2.953	30.66 ± 4.442	56.89 ± 1.799	36.67 ± 4.766

a Values are expressed as means ± SD (*n* = 3).

**Table 7:** IC_50_ values of standard diclofenac and eugenol derivatives on inhibition of albumin denaturation

Compounds	IC_50_ values [μM]
Standard diclofenac sodium	54.32
1A	236.0
1B	313.7
1C	133.8
1D	213.3

Albumin denaturation is a process leading to the loss of secondary and tertiary structure of proteins due to external stress such as strong acid or base, organic solvent, or heat. When biological proteins are denatured, they lose their biological function. Hence, a ligand's ability to inhibit the denaturation of protein signifies the potential for anti-inflammatory activity. The anti-inflammatory potential was depicted clearly in the albumin denaturation assay. The present research shows that newly synthesized compounds can limit the formation of autoantigens caused by protein denaturation and stabilize lysosomal membranes *in vivo*. This research provides the scientific groundwork for various inflammatory diseases. *In vivo* studies can also be applied to understand the mechanism of the anti-inflammatory activity of newly synthesized eugenol derivatives.

## 3. Summary and conclusions

The titled compounds were designed based on pharmacodynamics and pharmacokinetics requirements. The pharmacokinetics disclosed that newly synthesized compounds 1A–D obey Lipinski's rule and show promising drug scores. The docked structures at the binding sites were found to be stable using molecular dynamics simulations. The average RMSD of Cα atoms of PPAR-gamma and heavy atoms of 1C was found to be 2.490 ± 0.1105 Å compared to the standard drug pioglitazone with PPARγ complex had an average RMSD of 2.440 ± 0.07039 Å. The *R*_g_ values for protein–ligand complexes: PPAR gamma 1C and pioglitazone show fluctuations between 18.6 Å to 18.9 Å while, lysozyme bound complexes *R*_g_ values are between 13.65 Å to 13.85 Å. PPAR gamma 1D complex shows fewer deviations with time-dependent parameter analysis and compound 1C agrees with biological activity. We reported a simple, yet efficient method to synthesize some eugenol derivatives using substituted aromatic amines and characterized using spectroscopic techniques (FTIR, ^1^H NMR, ^13^C NMR, and mass spectrometry). TR-FRET assays validated our *in silico* prediction results. Compound 1C lysozyme complex shows the best results for anti-inflammatory activity. *In vitro* anti-inflammatory results show that the synthesized compounds at different concentrations of 6.25–400 μM significantly protected heat-induced albumin denaturation. Among four compounds tested 1C showed potent anti-inflammatory activity with an IC_50_ value of 133.8 μM compared with standard diclofenac with an IC_50_ value of 54.32 μM.

## 4. Experimental

### 4.1 General procedure for the synthesis of eugenol derivatives

#### 4.1.1. Step 1: Synthesis of substituted acylated amine

Substituted aromatic amine (1 equivalent) and triethylamine (1.05 equivalent) along with dichloromethane (80–100 ml) were transferred into a flask fitted with a guard tube. While the above mixture was stirred under iced conditions (0–5 °C), 1.05 equivalents of chloroacetyl chloride were added drop by drop for 30 minutes. The reaction mixture was again stirred for about 10–12 h at room temperature. The reaction was monitored with thin-layer chromatography (TLC) using *n*-hexane and ethyl acetate as the mobile phase. After completion of the reaction, the mixture was treated with water and dilute HCl and transferred to a separating funnel, and allowed to separate. The water layer was removed and the DCM layer was passed through anhydrous sodium sulfate. The solvent was evaporated to obtain acylated amines.

#### 4.1.2. Step 2: Coupling eugenol with substituted acylated amines

A mixture of acylated amines (1 eq.), finely powdered anhydrous potassium carbonate (K_2_CO_3_ 3 eq), eugenol (1.2 eq.) along with 80 ml of dry acetone was stirred at 45 °C for about 26 h. The reaction progress was monitored by checking the spots, from time to time during the reaction using TLC with *n*-hexane and ethyl acetate as a mobile phase. Acetone was evaporated and the reaction mixture was extracted with ethyl acetate. The ethyl acetate layer was washed three times with a 10% NaOH solution, once with water, and then with brine solution before being dried on anhydrous sodium sulfate as described in Scheme 1. The ethyl acetate layer was evaporated to obtain the final compound as illustrated in Table 8.

**Table 8:** Details of synthesized compounds^a^

Compound	Chemical structure	Mol. wt	Mol. formula	*R*_f_ value	% Yield
1A	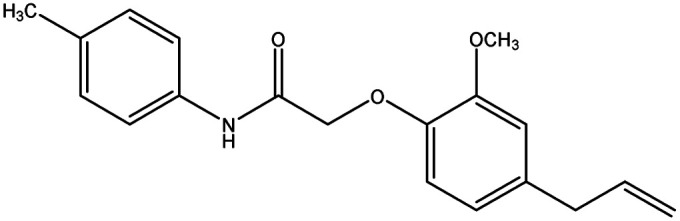	311.375	C_19_H_21_NO_3_	0.67	75.4
1B	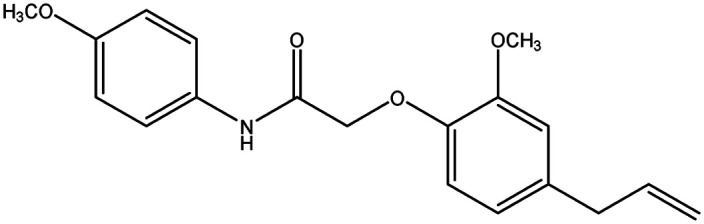	327.374	C_19_H_21_NO_4_	0.62	73.8
1C	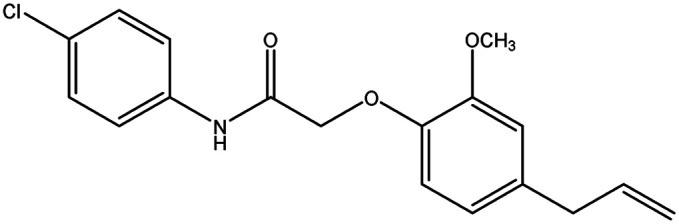	331.793	C_18_H_18_ClNO_3_	0.65	79.5
1D	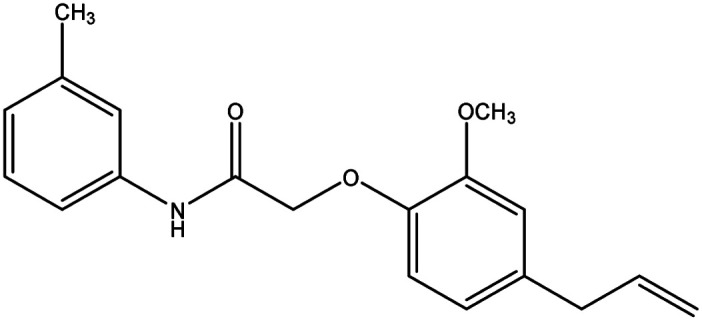	311.375	C_19_H_21_NO_3_	0.67	74.1

a The spectral data of all the newly synthesized eugenol derivatives.

#### 4.1.3. 2-(4-Allyl-2-methoxyphenoxy)-*N-p*-tolylacetamide (1A)

Colorless solid: yield: 75.4%, molecular formula: C_18_H_21_NO_3_. IR (KBr, cm^−1^): 3460.41 (N–H), 3074.63 (ArC–H), 2918.40 (AliC–H), 1681.98 (C=O), 1602.90 (ArC=C) and 1317.43 (C–O). ^1^H-NMR (400 MHz, *δ* ppm, CDCl_3_): 8.91 (s, 1H, NH), 7.48 (d, 2H, ArH, *J* = 8.0 Hz), 7.15 (d, 2H, ArH, *J* = 8.0 Hz), 6.92 (s, 1H, ArH), 6.77 (d, 2H, ArH, *J* = 9.6 Hz), 5.97 (m, 1H, =CH, *J* = 13.2 Hz), 5.11 (d, 2H, =CH_2_, *J* = 6.4 Hz), 4.61 (s, 2H, CH_2_), 3.91 (s, 3H, O–CH_3_), 3.35 (d, 2H, –CH_2_*J* = 6.8 Hz), 2.31 (s, 3H, –CH_3_). ^13^C-NMR: (400 MHz, *δ* ppm, CDCl_3_): 167.0 (C=O), 149.8 (C–O), 145.8 (O–C), 137.3 (–CH=), 135.8 (C–NH), 134.8 (ArC), 134.3 (ArC), 129.6 (2ArC), 121.2 (2ArC), 120.0 (ArC), 116.8 (=CH_2_), 116.1 (ArC), 112.7 (ArC), 70.9 (CH_2_–O), 56.0 (O–CH_3_), 40.0 (CH_2_), 21.0 (CH_3_). LC-MSMS (*m*/*z*) peak calculated for C_18_H_21_NO_3_ [M + 1]^+^ 312.1521 peak Found [M + 1]^+^ 312.1519.

#### 4.1.4. 2-(4-Allyl-2-methoxyphenoxy)-*N*-(4-methoxyphenyl) acetamide (1B)

Colorless solid: yield: 73.8%, molecular formula: C_18_H_21_NO_4_. IR (KBr, cm^−1^): 3282.95 (N–H), 3082.35 (ArC–H), 2914.54 (AliC–H), 1670.41 (C=O), 1595.18 (ArC=C) and 1336.71 (C–O). ^1^H-NMR (400 MHz, *δ* ppm, CDCl_3_): 8.83 (s, 1H, NH), 7.50 (d, 2H, ArH, *J* = 8.8 Hz), 6.92 (s, 1H, ArH) 6.90 (d, 1H, 2ArH *J* = 4.8 Hz), 6.77 (d, 2H, ArH, *J* = 8.8 Hz), 5.98 (m, 1H, =CH, *J* = 10.4 Hz), 5.11 (d, 2H, =CH_2_, *J* = 6.0 Hz), 4.62 (s, 2H, CH_2_), 3.91 (s, 3H, O–CH_3_), 3.79 (s, 3H, –OCH_3_) 3.36 (d, 2H, –CH_2_). ^13^C-NMR: (400 MHz, *δ* ppm, CDCl_3_): 166.7 (C=O), 156.5 (C–O), 149.7 (C–O), 145.7 (O–C), 137.1 (–CH=), 135.7 (C–NH), 130.4 (ArC), 121.5 (2ArC), 121.1 (ArC), 116.6 (=CH_2_), 116.0 (ArC), 114.1 (2ArC), 112.5 (ArC), 70.7 (CH_2_–O), 55.9 (O–CH_3_), 55.4 (O–CH_3_), 39.8 (CH_2_). LC-MSMS (*m*/*z*) peak calculated for C_18_H_21_NO_4_ [M + 1]^+^ 328.1471 peak found [M + 1]^+^ 328.1469.

#### 4.1.5. 2-(4-Allyl-2-methoxyphenoxy)-*N*-(4-chlorophenyl) acetamide (1C)

Colorless solid: yield: 79.5%, molecular formula: C_18_H_18_ClNO_3_. IR (KBr, cm^−1^): 3342.75 (N–H), 3078.49 (ArC–H), 2839.31 (AliC–H), 1693.56 (C=O), 1591.33 (ArC=C), 1307.78 (C–O) and 817.85 (C–Cl). ^1^H-NMR (400 MHz, *δ* ppm, CDCl_3_): 9.00 (s, 1H, NH), 7.56 (d, 2H, ArH, *J* = 8.8 Hz), 7.30 (d, 2H, ArH, *J* = 8.8 Hz), 6.92 (s, 1H, ArH), 6.77 (d, 2H, ArH, *J* = 8.8 Hz), 5.97 (m, 1H, =CH, *J* = 9.2 Hz), 5.11 (d, 2H, =CH_2_, *J* = 6.4 Hz), 4.61 (s, 2H, CH_2_), 3.91 (s, 3H, O–CH_3_), 3.35 (d, 2H, –CH_2,_*J* = 6.8 Hz). ^13^C-NMR: (400 MHz, *δ* ppm, CDCl_3_): 172.1 (C=O), 154.8 (C–O), 150.7 (O–C), 142.1 (C–NH), 141.0 (–CH=), 134.5 (ArC), 134.1 (2ArC), 126.2 (C–Cl), 126.0 (2ArC), 121.1 (2ArC), 117.7 (=CH_2_), 75.9 (CH_2_–O), 61.0 (O–CH_3_), 44.9 (CH_2_). LC-MSMS (*m*/*z*) peak calculated for C_18_H_18_ClNO_3_. [M + 1]^+^ 332.0975 peak found [M + 1]^+^ 332.0973.

#### 4.1.6. 2-(4-Allyl-2-methoxyphenoxy)-*N-m*-tolylacetamide (1D)

Dark brownish black liquid: yield: 74.1%, molecular formula: C_18_H_21_NO_3_. IR (KBr, cm^−1^): 3333.10 (N–H), 3076.56 (ArC-H), 2935.76 (AliC-H), 1687.77 (C=O), 1595.18 (ArC=C) and 1377.22 (C–O). ^1^H-NMR (400 MHz, *δ* ppm, CDCl_3_): 8.97 (s, 1H, NH), 7.48 (s, 1H, ArH), 7.41 (d, 1H, ArH), 6.96 (d, 2H, ArH, *J* = 7.6 Hz), 6.86 (t, 1H, ArH, *J* = 8.4 Hz), 6.79 (d, 2H, ArH, *J* = 5.6 Hz), 6.69 (s, 1H, ArH), 6.00 (m, 1H, =CH, *J* = 13.6 Hz), 5.13 (d, 2H, =CH_2_, *J* = 8.4 Hz), 4.62 (s, 2H, CH_2_), 3.92 (s, 3H, O–CH_3_), 3.37 (d, 2H, –CH_2,_*J* = 6.4 Hz) 2.36 (s, 3H, CH_3_). ^13^C-NMR: (400 MHz, *δ* ppm, CDCl_3_): 172.1 (C=O), 149.0 (C–O), 143.9 (O–C), 142.3 (C–NH), 136.9 (–CH=), 133.9 (ArC), 130.4 (ArC), 126.2 (ArC), 125.6 (ArC), 122.0 (ArC), 121.7 (ArC), 121.1 (ArC), 119.5 (ArC), 117.6 (=CH_2_), 116.3 (ArC), 75.8 (CH_2_–O), 60.9 (O–CH_3_), 44.9 (CH_2_), 26.5 (-CH_3_). LC-MSMS (*m*/*z*) peak calculated for C_18_H_21_NO_3_. [M − 1] 310.1521 peak found [M − 1] 310.1520.

### 4.2 ADMET, TOPKAT and drug likeness

The pharmacokinetics and dynamics properties of compounds were analyzed through a small molecular protocol (BIOVIA, Discovery Studio 2019) to understand the molecular behavior. Further, mutagenicity and carcinogenicity, and the set dosage range of the compounds were analyzed using the Bayesian and regression model. In addition, Lipinski's rule of 5 was carried out to find out the oral bioavailability of the compounds.

### 4.3 Molecular docking

The structure of drug target protein and compounds employed for docking was prepared using the macromolecule tool and small molecule protocol in Discovery studio 2019. The bound ligand coordinates 49.720*X* −36.98*Y* 19.294*Z* of radius 8.4 Å for PPAR-Gamma (2PRG) and 26.63*X* 5.50*Y* 14.22*Z* of radius 12 Å for egg lysozyme (3WXU) with an equal grid spacing of 0.5 Å with 90-degree grid angles is defined as binding sites for docking. CDOCKER algorithm was employed to study receptor–ligand interaction, as it is a powerful CHARMm-based docking approach that has been demonstrated to produce extremely accurate docked poses. All the parameters were set as default while executing the docking process. The best poses are further probed for binding energy calculation with implicit solvent model PBSA. The energetically stable complex was taken for molecular dynamics simulation and nonbonded interaction analysis.^34–36^

### 4.4 Molecular dynamics simulation

The standard drugs and top two compounds of interaction with drug targets PPAR-gamma and egg lysozyme from the docking study were subjected to 1000 picosecond of molecular dynamics simulations using Dassault Systems BIOVIA, Discovery Studio 2019 Modeling Environment. It was carried out in five cascade steps beginning with two steps of 500 cycles of energy minimization of the complex with the steepest descent and conjugate gradient. Following heating, the system gradually forms 50 K to 300 K with 100 ps of simulation time and equilibration for 500 ps to attain degrees of freedom. Finally, the production with the canonical ensemble was subjected to equal *T*_mass_ and *P*_mass_ at 300 K. Additionally, the SHAKE constraint fixes all bonds involving hydrogen in the simulation, within this cutoff distance of 12–10 Å. All the atom velocities and positions are calculated at time points using the leap-frog verlet algorithm. At last, the deviations in the conformation of the complexes were analyzed by RMSD and *R*_g_.

### 4.5 TR-FRET competitive ligand displacement assay

We performed a time-resolved fluorescence resonance energy transfer (TR-FRET) assay using a black flat bottom 384-well plate and a buffer containing 20 mM potassium phosphate (pH 7.4), 0.5 mM EDTA, 50 mM KCl, and 0.01% Tween-20, 5 mM TCEP. For the TR-FRET coregulator interaction assay, each well contained 400 nM FITC-labeled TRAP220 or NCoR1 peptides, 1 nM LanthaScreen Elite Tb-anti-His Antibody, 4 nM 6xHis-PPARγ LBD protein and 400 nM peptide in TR-FRET buffer in 22.5 μL total volume per well. Each well (22.5 L per well) included 1 nM 6xHis-PPAR LBD protein, 1 nM LanthaScreen Elite Tb-anti-His Antibody, and 5 nM Fluormone Pan-PPAR Green tracer ligand in TR-FRET buffer for the ligand displacement experiment. Ligand stocks were made by serial dilution in DMSO, then added to wells in triplicate to a final DMSO concentration of 1%, then incubated at room temperature for 1 hour, and read using a BioTek Synergy Neo multimode plate reader. The Tb donor was stimulated at 340 nm, its fluorescence emission was measured at 490 nm, and the FITC emission of the acceptor was detected at 520 nm. The signal at 520 nm/490 nm was used to compute the TR-FRET ratio.^37,38^

### 4.6 Anti-inflammatory activity

#### 4.6.1. Albumin denaturation assay

The reaction mixture containing 0.2 ml of egg albumin (from fresh hen's eggs), 2.8 ml of phosphate-buffered saline (pH 6.4), and were mixed with 2 ml of the synthesized compounds in varying concentrations. Then the samples were incubated at 37 °C for 20 min and then heated to 70 °C for 20 min. After cooling the samples, the turbidity was measured spectrophotometrically at 660 nm. Diclofenac sodium was used as a reference standard. The following formula was used to calculate the percentage inhibition of protein denaturation.
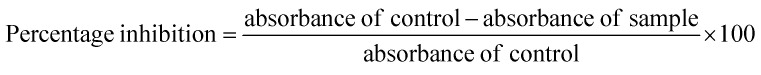


## Abbreviations

RT: Room temperature
TLC: Thin-layer chromatography
DMSO: Dimethylsulfoxide
Cox-2: Cyclooxygenase-2
IL-6: Interleukin-6
IL-1β: Interleukin-1 beta
iNOS: Inducible nitric oxide synthase
NF-Κb: Nuclear factor-kappa B
NO: Nitric oxide
PGE: Prostaglandins
TNF-α: Tumor necrosis factor α
ROS: Reactive oxygen species

## Author contributions

Noor Fathima Anjum and Prashantha Kumar BR have performed the synthesis and analysis of the reported compounds. Madhusudan N Purohit conceived the idea and planning. Dhivya Shanmugarajan, Syed Faizan, and Namburu Lalitha Naishima performed the rational drug design and analysis. Saleem Javid and Vasanth Kumar Shivaraju have performed the TR-FRET and anti-inflammatory studies.

## Conflicts of interest

The authors declare no conflict of interest.

## Supplementary Material

RA-012-D2RA02116A-s001
