# Supplementary material for: Novel derivatives of eugenol as potent anti-inflammatory agents via PPARγ agonism: rational design, synthesis, analysis, PPARγ protein binding assay and computational studies
Source: RSC Adv. 2022 Jun 7;12(26):16966–78. doi: 10.1039/d2ra02116a (PMC9172550; doi:10.1039/d2ra02116a)
Supplement: RA-012-D2RA02116A-s001 [file RA-012-D2RA02116A-s001.pdf]

Electronic Supplementary Material (ESI) for RSC Advances.
This journal is © The Royal Society of Chemistry 2022

Novel derivatives of eugenol as potent anti-inflammatory agents via PPARγ agonism: Rational design, synthesis, analysis, PPARγ protein binding assay, and computational studies

Noor Fathima Anjum[a], Dhivya Shanmugarajan[b], Vasanth Kumar Shivaraju,[c] Syed Faizan[b], Namburu Lalitha Naishima [b], Prashantha Kumar BR[b], Saleem Javid[a], Madhusudan N. Purohit[b] *

[a]*Department of Pharmaceutical Chemistry, Farooqia College of Pharmacy, Mysuru 570 015, India.*

[b]*Department of Pharmaceutical Chemistry, JSS College of Pharmacy, Mysuru 570 015, India. JSS Academy of Higher Education & Research, Mysuru 570 015, India.*

[c]*DOS in Chemistry, Karnataka State Open University, Mukthagangothri, Mysuru 570 006, India.*

Supplementary Material

CONTENTS **Page No.**

1. **Spectra……………………………………………………….............. 2-9**
2. **TR-FRET Assay……………………………………………………… 10-11**

Spectra of synthesized compounds

1. **IR, ^{H}NMR, ^{13}CNMR & Mass Spectra of compound 1A**

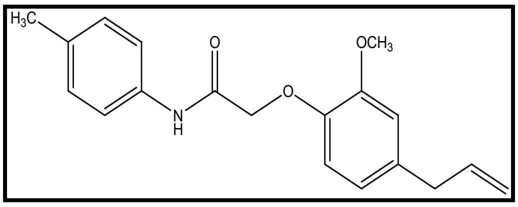

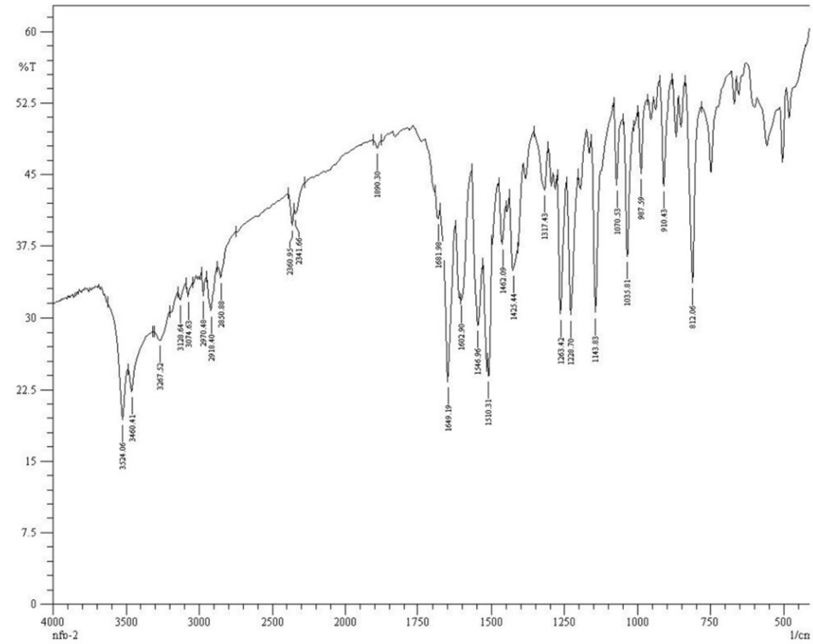

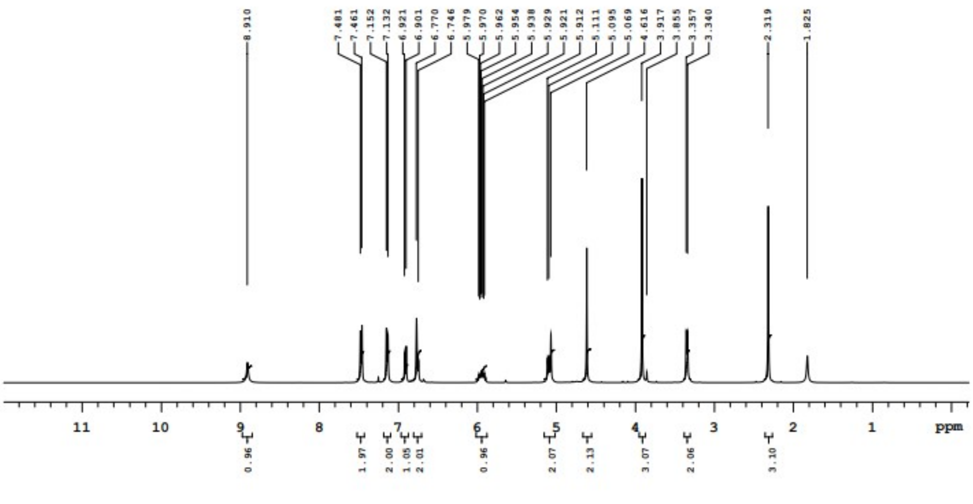

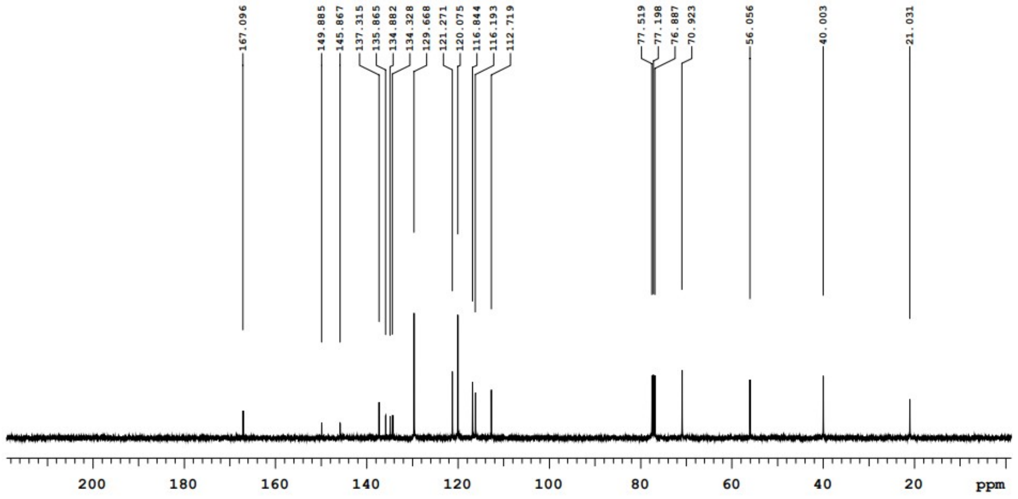
167.096
149.885
145.867
137.315
135.865
134.882
134.328
129.668
121.271
120.075
116.844
116.193
112.719
77.519
77.198
76.887
70.923
56.056
40.003
21.031
200
180
160
140
120
100
80
60
40
20
ppm

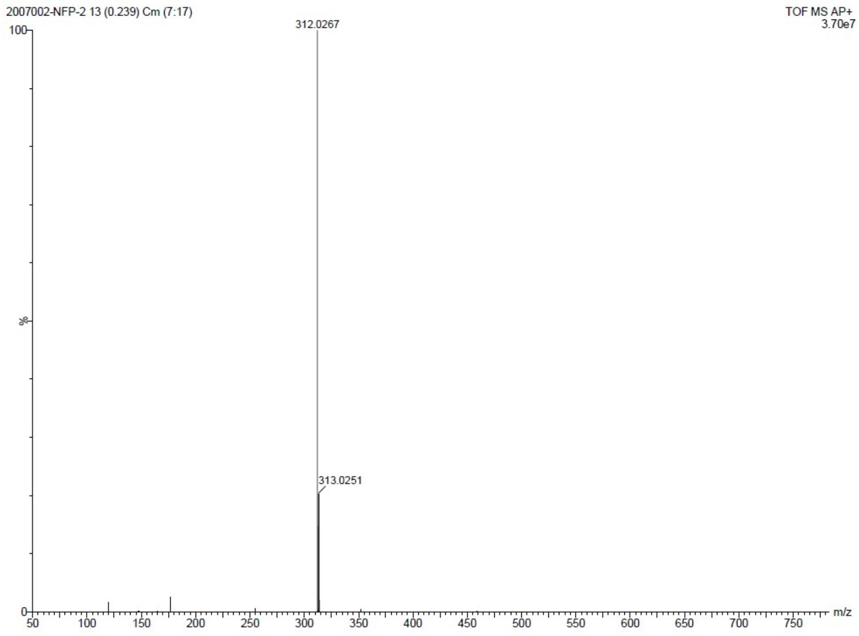
2007002-NFP-2 13 (0.239) Cm (7:17)
TOF MS AP+
3.70e7
312.0267
313.0251
100
%
0
50
100
150
200
250
300
350
400
450
500
550
600
650
700
750
m/z

2. IR, HNMR, 13CNMR & Mass Spectra of compound 1B

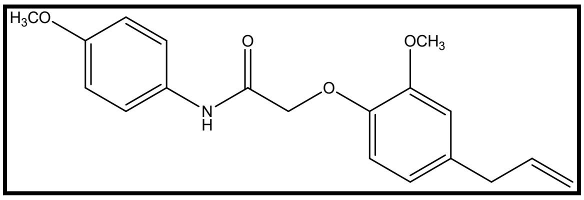

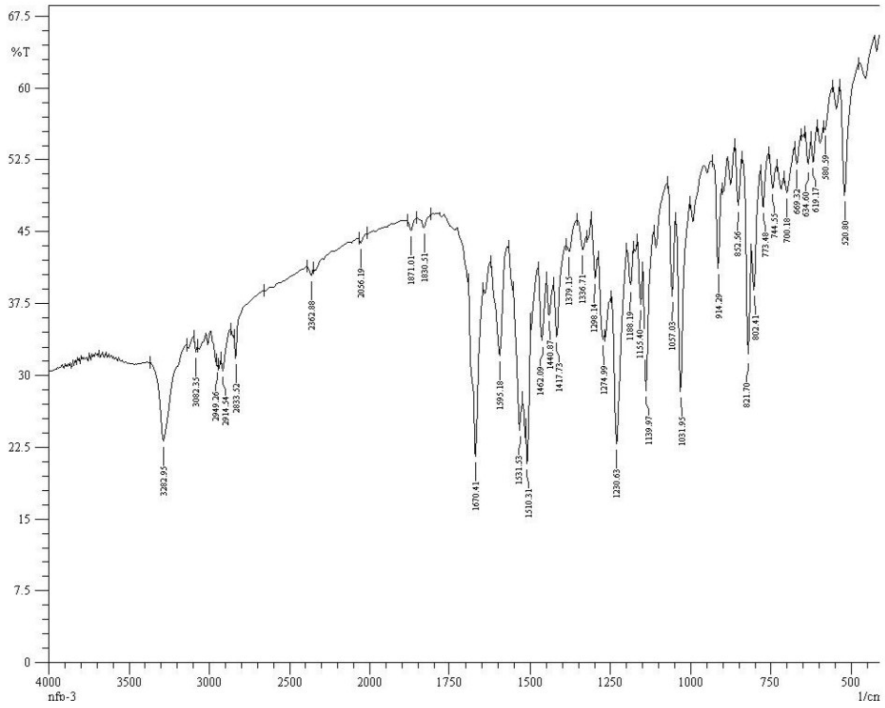

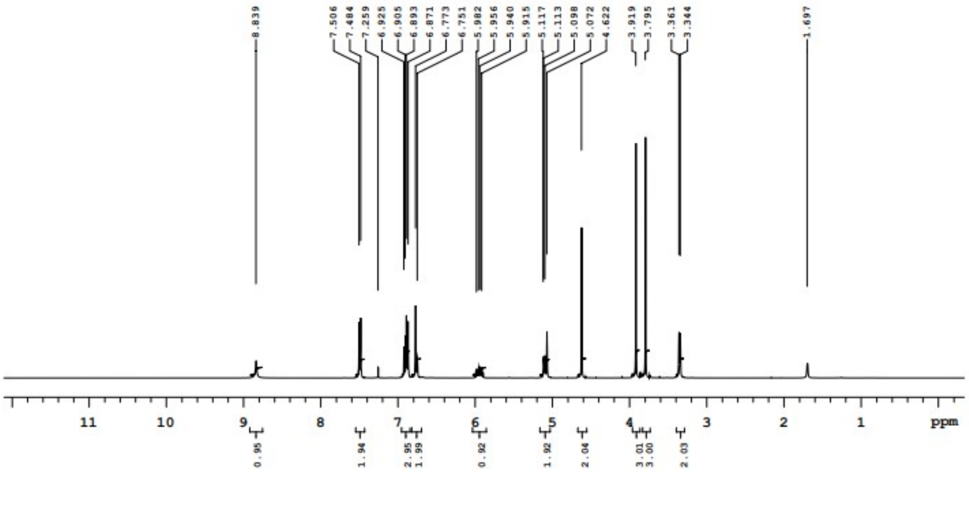

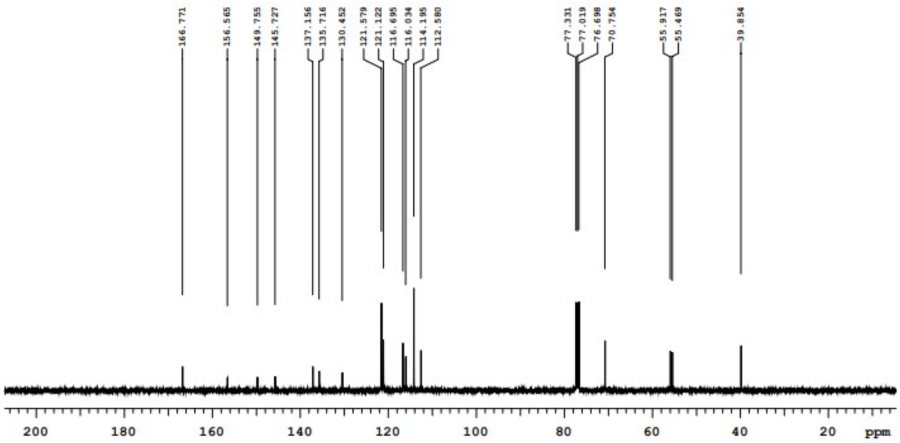
166.771
156.565
149.755
145.727
137.156
135.716
130.452
121.579
121.122
116.695
116.034
114.195
112.580
77.331
77.019
76.698
70.754
55.917
55.469
39.854
200
180
160
140
120
100
80
60
40
20
ppm

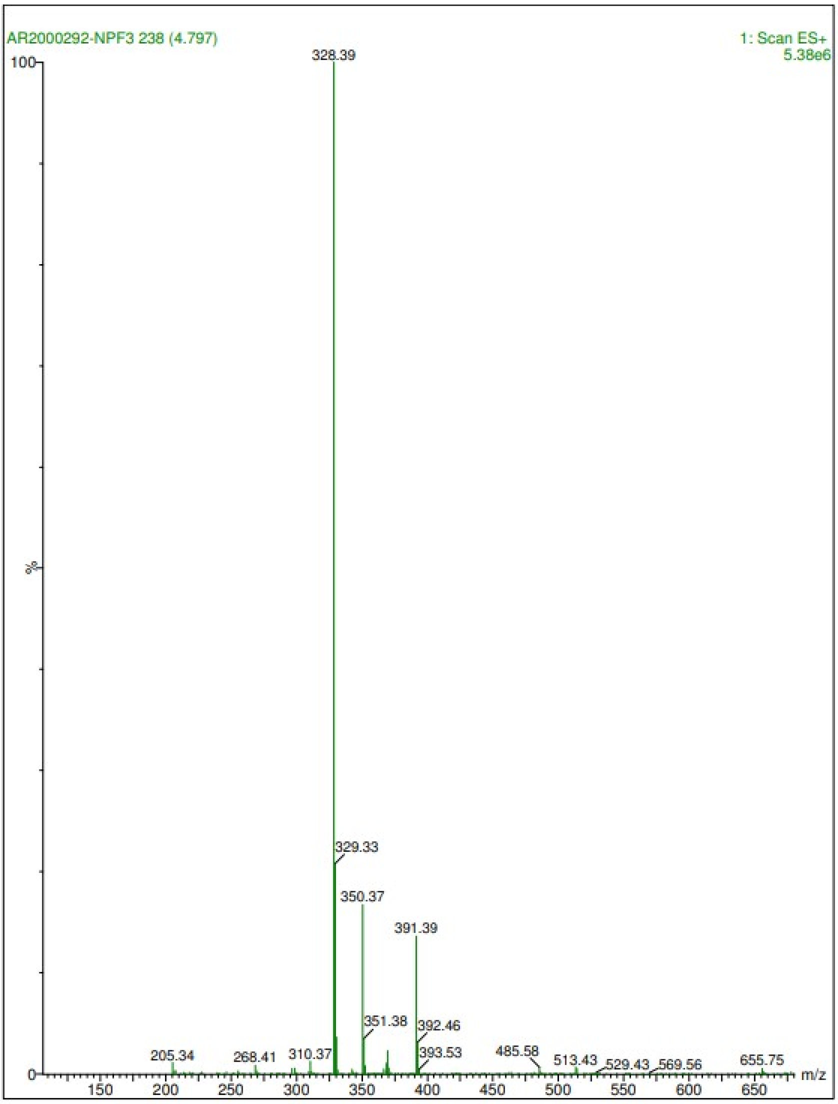
AR2000292-NPF3 238 (4.797)
1: Scan ES+
5.38e6
100
%
0
328.39
329.33
350.37
391.39
351.38
392.46
393.53
205.34
268.41
310.37
485.58
513.43
529.43
569.56
655.75
150
200
250
300
350
400
450
500
550
600
650
m/z

3. IR, ^{H}NMR, ^{13}CNMR & Mass Spectra of compound 1C

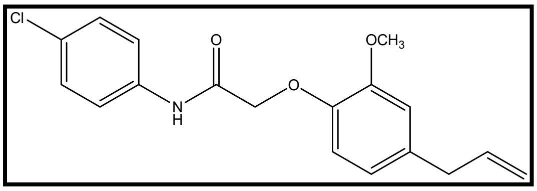

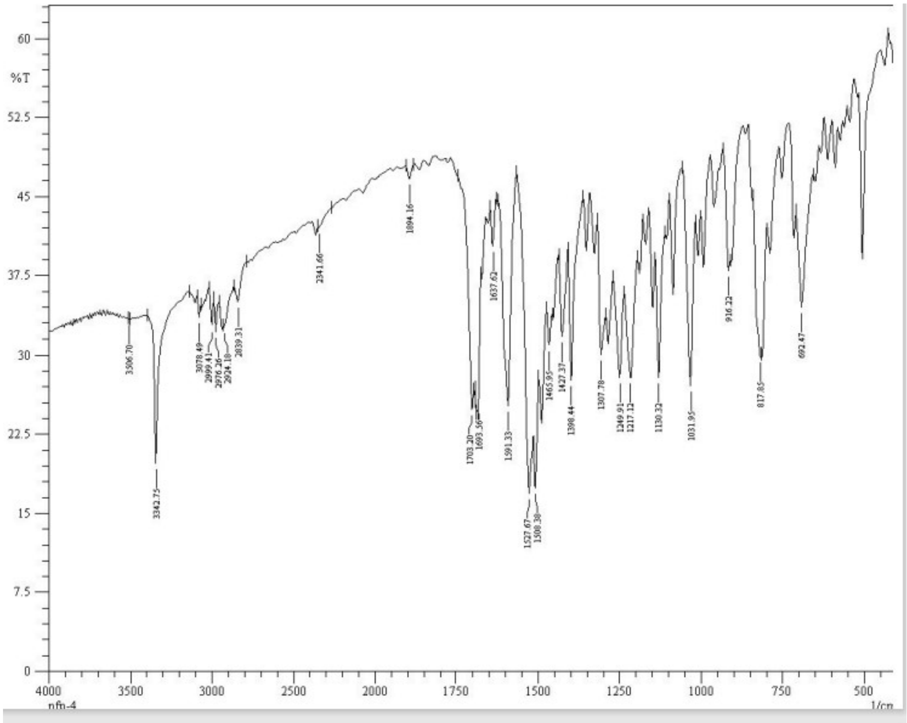

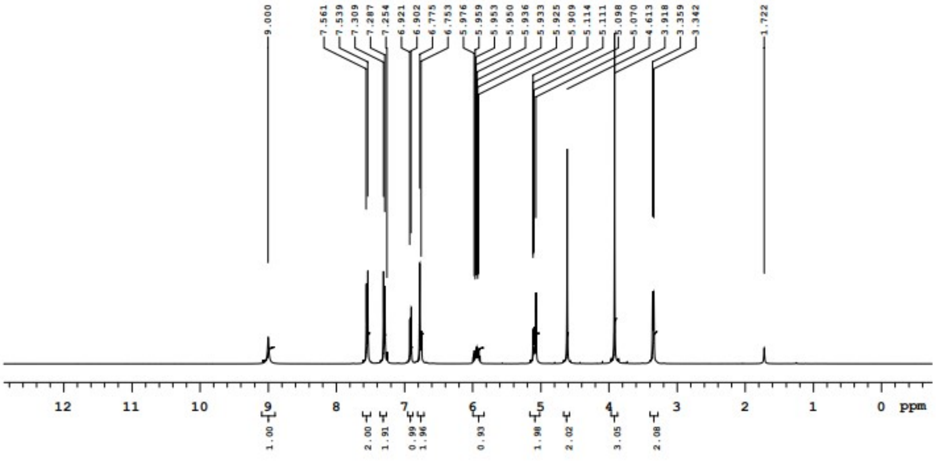

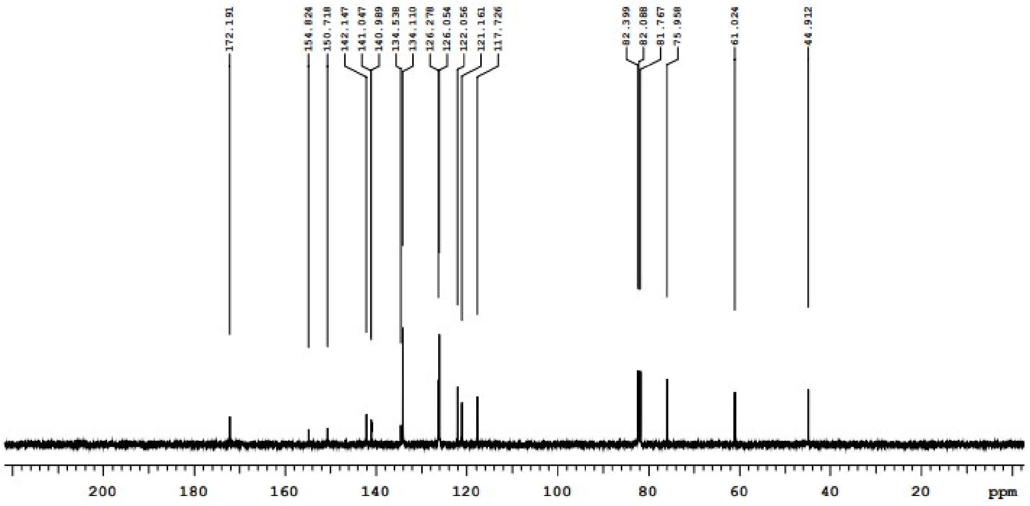

172.191
154.824
150.718
142.147
141.047
140.989
134.538
134.110
126.278
126.054
122.056
121.161
117.726
82.399
82.088
81.767
75.958
61.024
44.912
200
180
160
140
120
100
80
60
40
20
ppm

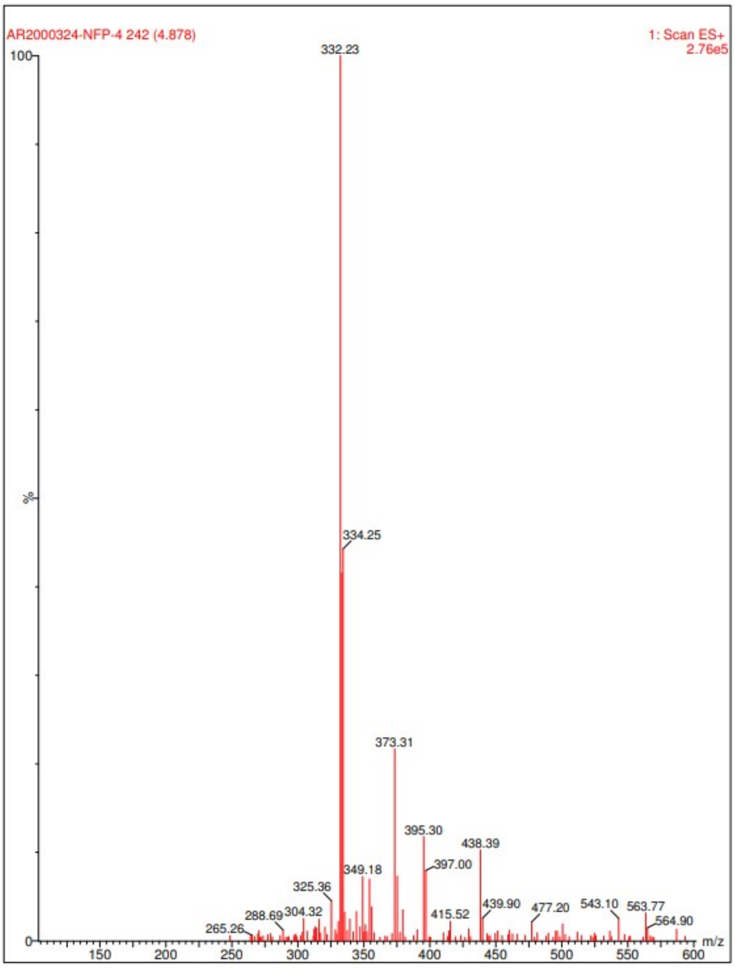

AR2000324-NFP-4 242 (4.878)
1: Scan ES+
2.76e5
100
%
0
332.23
334.25
373.31
395.30
438.39
397.00
349.18
325.36
304.32
288.69
265.26
415.52
439.90
477.20
543.10
563.77
564.90
150
200
250
300
350
400
450
500
550
600
m/z

3. IR, ^{H}NMR, ^{13}CNMR & Mass Spectra of compound 1D

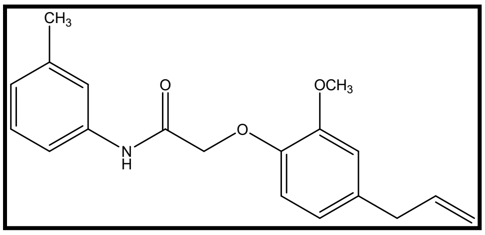

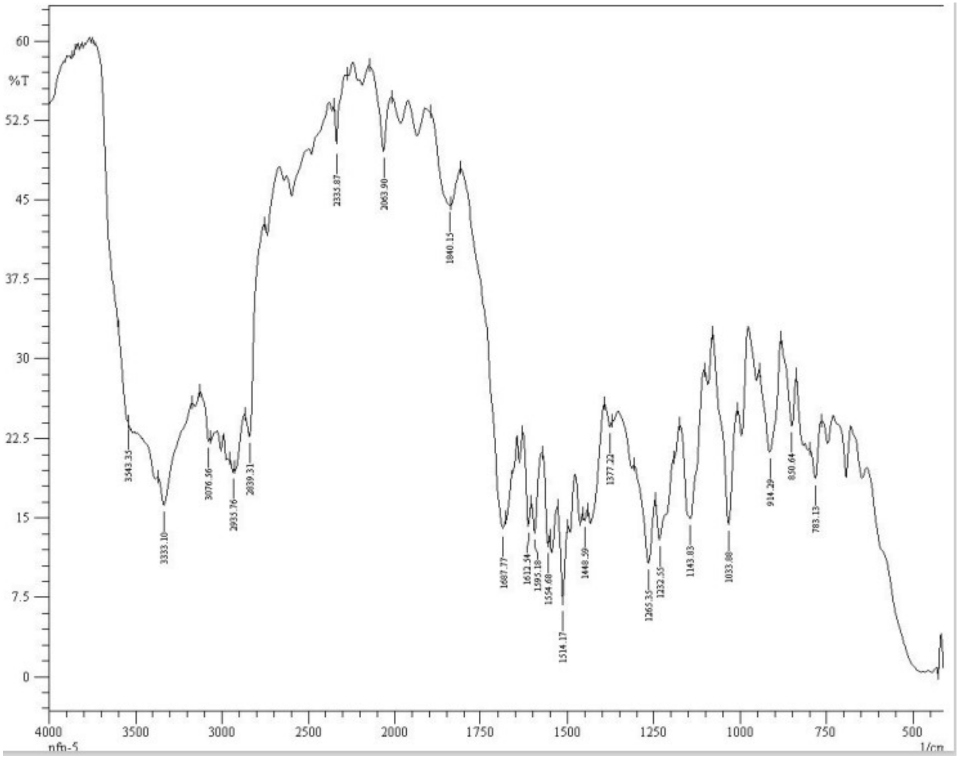

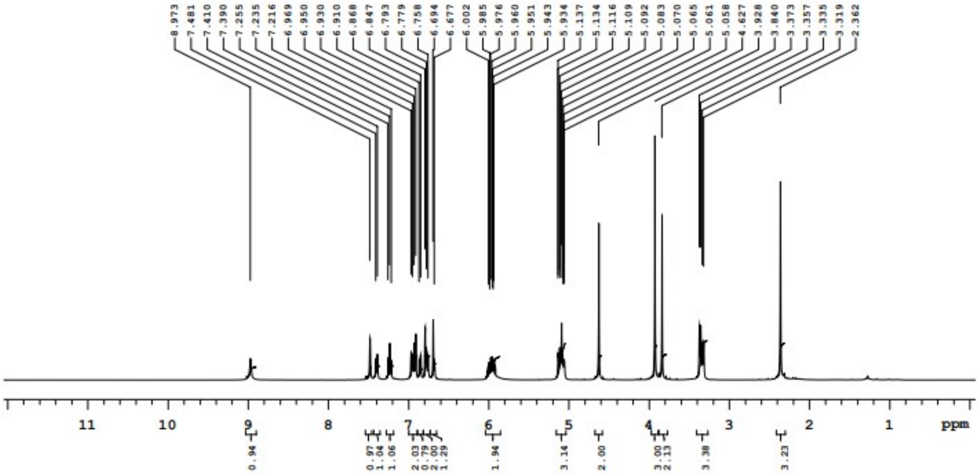

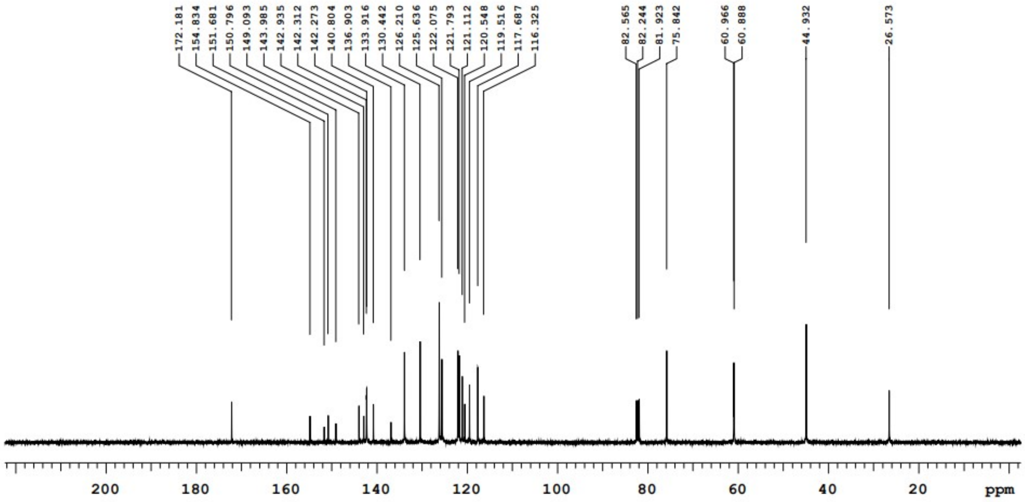
172.181
154.834
151.681
150.796
149.093
143.985
142.935
142.312
142.273
140.804
136.903
133.916
130.442
126.210
125.636
122.075
121.793
121.112
120.548
119.516
117.687
116.325
82.565
82.244
81.923
75.842
60.966
60.888
44.932
26.573
ppm

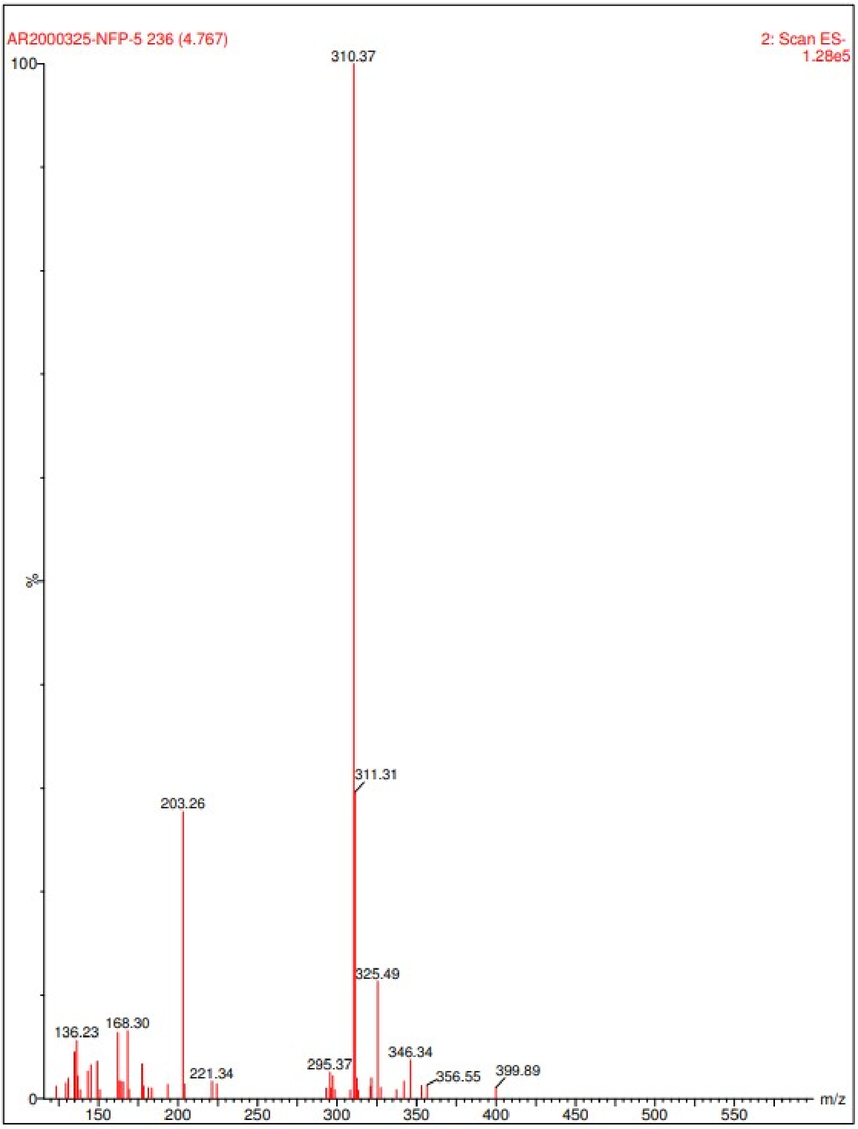
AR2000325-NFP-5 236 (4.767)
2: Scan ES-
1.28e5
310.37
311.31
203.26
325.49
136.23
168.30
295.37
346.34
221.34
356.55
399.89
%
m/z

TR-FRET Assay:

Compound 1A

SL No.	1A (Concentration (µM)	Raw Data (RLU)	Raw Data - Background (RLU)
1	100	0.54	0.300619419
2	20	1.07	0.839901744
3	4	1.57	1.33177375
4	0.8	1.99	1.754049283
5	0.16	1.95	1.715545715
6	0.032	2.00	1.760204347
7	0.0064	1.92	1.687764768
8	0.00128	2.02	1.787422152
9	0.000256	2.10	1.861679618
10	0.0000512	2.07	1.839505412

Compound 1B

SL No.	1B Concentration (µM)	Raw Data (RLU)	Raw Data - Background (RLU)
1	100	0.534	0.298879758
2	20	1.280	1.04507794
3	4	1.855	1.619466248
4	0.8	2.002	1.767370589
5	0.16	1.960	1.724473418
6	0.032	1.913	1.678017542
7	0.0064	1.913	1.678286208
8	0.00128	1.965	1.73005786
9	0.000256	1.990	1.754676693
10	0.0000512	2.024	1.788731767

Compound 1C

SL No.	1C Concentration (µM)	Raw Data (RLU)	Raw Data - Background (RLU)
1	100	0.951	0.716
2	20	1.058	0.823
3	4	1.761	1.526
4	0.8	1.922	1.687
5	0.16	1.921	1.686
6	0.032	1.989	1.754
7	0.0064	1.934	1.699
8	0.00128	1.907	1.672
9	0.000256	1.896	1.660
10	0.0000512	2.069	1.834

Compoundd 1D

SL No.	1D (Concentration (µM)	Raw Data (RLU)	Raw Data - Background (RLU)
1	100	1.40	1.17
2	20	1.91	1.67
3	4	1.91	1.67
4	0.8	1.91	1.68
5	0.16	1.87	1.64
6	0.032	1.93	1.70
7	0.0064	2.07	1.83
8	0.00128	1.85	1.61
9	0.000256	1.95	1.72
10	0.0000512	1.92	1.69
